# CYP2C19 genotyping and mavacamten: predicting outcomes in normal, intermediate and rapid metabolisers in obstructive hypertrophic cardiomyopathy

**DOI:** 10.1007/s00228-025-03991-8

**Published:** 2026-02-06

**Authors:** Yande Kasolo, Edward Burford, Mohammed Obeidat, Glenda M Beaman, Thomas Monk, Rachel Bastiaenen, William G Newman, Robert M Cooper

**Affiliations:** 1 Liverpool Heart and Chest Hospital NHS Foundation Trust, Liverpool, UK; 2 Inherited Cardiovascular Conditions Group, Guy’s and St Thomas NHS Foundation Trust, London, UK; 3 British Heart Foundation Centre of Research Excellence, School of Cardiovascular and Metabolic Medicine and Sciences, King’s College London, London, UK; 4 Division of Cardiovascular Sciences, University of Manchester, Manchester, UK; 5 Division of Evolution, Infection and Genomics, School of Biological Sciences, University of Manchester, Manchester, M13 9PT UK; 6 Manchester Centre for Genomic Medicine, St Mary’s Hospital, Manchester University NHS Foundation Trust, Manchester, M13 9WL UK; 7 North West Genomic Laboratory Hub, Manchester University NHS Foundation Trust, Manchester, M13 9WL UK; 8 Research Institute of Sports and Exercise Science, Liverpool John Moores University, Liverpool, UK; 9 Liverpool Heart and Chest Hospital NHS Foundation Trust, Thomas Drive, Liverpool, Merseyside, L14 3PE UK

**Keywords:** Hypertrophic cardiomyopathy, CYP2C19, Mavacamten, Genotyping

## Abstract

**Purpose:**

Mavacamten is the first targeted therapy for obstructive hypertrophic cardiomyopathy (oHCM). It is metabolised via cytochrome p450 enzymes, with variations in the *CYP2C19* gene having predominant influence on plasma concentrations of mavacamten. We aimed to outline the effect of CYP2C19 metaboliser status on outcomes in patients taking mavacamten.

**Methods:**

We retrospectively analysed clinical and echocardiographic data in patients with symptomatic oHCM taking mavacamten. *CYP2C19* genotyping was undertaken by loop-mediated isothermal amplification (LAMP) on EDTA whole blood (LaCAR MDx, Liege Belgium) followed by Sanger sequencing of the coding exons of *CYP2C19*. Logistical regression was used to assess time taken to optimisation.

**Results:**

Fifty-five patients (59±13 years; 73% male) were included. Genotyping of CYP2C19*2, CYP2C19*3, and CYP2C19*17 alleles was conducted. Due to low numbers in the ultrarapid (*n* = 1) and poor (*n* = 2) groups, statistical analysis was performed in intermediate, normal and rapid metabolisers. Using normal metabolisers as the reference, there was a non-significant trend towards faster optimisation in intermediate metabolisers (odds ratio 0.63 [95% CI: 0.12–3.19]) and rapid metabolisers (OR 0.55 [95% CI: 0.11–2.53]). While reductions in peak resting (40 ± 34.37 mmHg) and Valsalva (64 ± 35.23 mmHg) left ventricular outflow tract gradients were statistically significant across the cohort (*p* < 0.0001), there was no interaction between differing CYP2C19 groups and time (*p* = 0.69).

**Conclusion:**

Excluding poor metabolisers, variations in the *CYP2C19* gene do not explain different clinical outcomes in patients with oHCM on mavacamten. Beyond genotyping of the targeted variants, *CYP2C19* sequencing did not provide any additional clinically relevant information.

**Supplementary Information:**

The online version contains supplementary material available at 10.1007/s00228-025-03991-8.

## Introduction

Hypertrophic Cardiomyopathy (HCM) is a condition characterised by abnormal thickening of the left ventricular myocardium. It has a prevalence of at least 1 in 500 individuals and is most commonly caused by mutations in sarcomeric genes [1]. In HCM, there is upregulation of actin-myosin cross bridging in the cardiac myocytes, resulting in a hypercontractile state [2]. Dynamic left ventricular outflow tract (LVOT) obstruction is a core pathophysiological feature of HCM often resulting in symptoms such as chest pain, dyspnoea and exercise limitation [3]. Beta blockers, non-dihydropyridine calcium channel blockers and disopyramide have historically been used to treat LVOT obstruction [4, 5]. However, these non-targeted therapies are often poorly tolerated, with dose titration limited by adverse side effects [6, 7]. Invasive septal reduction therapies such as alcohol septal ablation and surgical myectomy improve long term survival and symptoms in patients with drug-resistant presentations [8]. However, these invasive procedures require centre-specific expertise and may not be appropriate in all patients.

Mavacamten, a first-in class, allosteric inhibitor of cardiac myosin ATPase, targets the underlying hypercontractile physiology of HCM [9]. It is effective in reducing symptoms and improving exercise capacity, as demonstrated in phase III randomised controlled trials [10, 11]. In EXPLORER-HCM, significant reduction in LVOT gradient was seen at 30 weeks, with a 35.6mmHg greater mean reduction in peak post-exercise gradient compared with placebo (95% CI − 43·2 to − 28·1; *p* < 0·0001). 37% of patients on mavacamten met the primary endpoint, a composite of improved New York Heart Association (NYHA) symptom class and peak oxygen uptake (pVO_2_) (*p* < 0.0005) [10]. 

The estimated oral bioavailability for mavacamten is at least 85%, with a rapid median time to maximum concentration (around 1 h) [9]. It is metabolised via the liver, predominantly through cytochrome p450 enzymes CYP2C19, CYP3A4 and CYP2C9. CYP2C19 is responsible for 74% of its metabolism [12, 13]. Individual variants in the *CYP2C19* gene lead to variation in mavacamten exposure, with five different metaboliser phenotypes reported: poor, intermediate, normal, rapid and ultrarapid [14]. Elimination half-life varies between these phenotypes: 6 days for ultrarapid metabolisers, 8 days for rapid metabolisers, 9 days for normal metabolisers, 10 days for intermediate metabolisers and 23 days for poor metabolisers [15]. 

In Europe and the United Kingdom (UK), the summary of product characteristics for mavacamten states that patients should be genotyped for *CYP2C19* to determine the appropriate dose. Poor metabolisers or those within unknown metaboliser status start on a lower dose of 2.5 mg rather than 5 mg once daily. The European Medicines Agency outlines a strict dosing regimen, which is separated into a 12 week initiation phase, dose titration and a subsequent maintenance phase. Clinical review with echocardiography occurs at 4 weekly intervals during the first 12 weeks. Dose reduction or temporary cessation of mavacamten occurs if peak LVOT gradient drops to < 20mmHg, or if the left ventricular ejection fraction (LVEF) falls below 50%. Beyond this period, patients are titrated up to a maximal dose of 5 mg (poor metabolisers) or 15 mg in other metaboliser groups, with a target LVOT gradient < 30mmHg and symptom resolution [15]. Currently in the UK, individuals are genotyped for the CYP2C19*2, CYP2C19*3 and CYP2C19*17 alleles to determine their metaboliser status. This practice differs from that in North America, where *CYP2C19* genotyping is not performed and hence does not inform dosing decisions. Given the time and cost incurred to facilitate genetic testing for these patients, assessment of its utility is an important avenue to explore.

## Aims and methods

We sought to determine the effect of CYP2C19 metaboliser status on outcomes in patients with obstructive HCM (oHCM) on mavacamten in two cardiomyopathy centres in the UK.

### Study design and setting

Consecutive patients with symptomatic oHCM treated with mavacamten from two UK centres (Guy’s and St Thomas’ Hospitals and Liverpool Heart and Chest Hospital) were included in this retrospective study. Data from baseline, week 4, week 8, week 12 and the most recent visit was collated. This included dose, echocardiographic parameters and New York Heart Association (NYHA) class. Initiation dose was dictated by metaboliser status, with poor metabolisers starting on 2.5 mg and other metaboliser groups starting on 5 mg, as per the European summary of product characteristics.

### Patient selection

Patients met established eligibility criteria for mavacamten therapy; including NYHA II-III symptoms, peak LVOT gradient ≥ 50mmHg and left ventricular ejection fraction (LVEF) ≥ 55%. Baseline characteristics are highlighted below in Table 1.

**Table 1:** Baseline characteristics of patient cohort on Mavacamten

	*N* = 55 (SD or %)
**Age**	59 (±13)
**Sex**	
Male	40 (73)
Female	15 (27)
**Ethnicity**	
White (British/European)	46 (84)
Black (African/Caribbean)	4 (7)
Asian	2 (4)
Other	3 (5)
**CYP2C19 metaboliser status**	
Ultrarapid	1 (2)
Rapid	16 (29)
Normal (extensive)	21 (38)
Intermediate	15 (27)
Poor	2 (4)
**HCM Genotype**	
Pathogenic variant in sarcomeric gene	10 (18)
No pathogenic variant identified	33 (60)
Variant of unknown significance	4 (7)
HCM genetic panel not tested/outcome awaited	8 (15)
**Prior HCM treatment**	
Beta blocker	46 (84)
Non-DHP calcium channel blockers	8 (15)
Disopyramide	24 (44)
Previous SRT	4 (7)
**NYHA class at baseline**	
I	0
II	24
III	31
IV	0
**NYHA at most recent follow up**	
I	35
II	17
III	3
IV	0

### Genotyping

Patients had *CYP2C19* genotyping for the CYP2C19*2, CYP2C19*3, and CYP2C19*17 alleles, undertaken by loop-mediated isothermal amplification (LAMP) on EDTA whole blood (LaCAR MDx, Liege Belgium) as per manufacturer’s instructions at baseline and dosing in the drug initiation phase was determined by this. Poor metabolisers were commenced on 2.5 mg daily, with other phenotypes starting on 5 mg. Sanger sequencing of the nine coding exons and exon/intron boundaries of *CYP2C19* (NM_000769.4) was undertaken (primers table S1) on extracted DNA as described previously [16]. 

### Statistical analysis

Patients were considered optimised once treatment entered the maintenance phase and no further dose titration was required, conventionally when the LVOT gradient was < 30mmHg. Descriptive statistics were used to outline baseline characteristics. Logistical regression was performed to assess whether CYP2C19 metaboliser status predicted time to optimisation. Change in ejection fraction (EF) and LVOT gradient was derived using two-way repeated measures ANOVA.

## Results

55 patients (59±13 years; 73% male) commenced on mavacamten between December 2023 and September 2024 were included. CYP2C19 metaboliser status was confirmed in the initiation phase (Table 1). Treatment was permanently discontinued in 2 patients due to non-adherence.

### Genetics

Genotyping of the CYP2C19*2, CYP2C19*3, and CYP2C19*17 alleles was successfully conducted for all 55 individuals (Table 2). The genotypes are consistent with published allele frequencies [17]. Additional Sanger sequencing of *CYP2C19* identified no additional rare or novel variants and the CYP2C19 metaboliser status was not altered for any individual compared to that determined by the genotyping of the three functional alleles.

**Table 2:** Frequencies of *CYP2C19* genotypes and predicted metaboliser status

CYP2C19 Genotype	Metaboliser status	*N* = 55 (%)	CPIC CYP2C19 Approximate genotype frequencies %
*1/*1	Extensive (normal)	21 (38)	39
*1/*2	Intermediate	11 (20)	18
*1/*17	Rapid	16 (29)	27
*2/*17	Intermediate	4 (7)	6
*2/*2	Poor	2 (4)	2
*17/*17	Ultrarapid	1 (2)	4

### Time taken to optimisation

44 patients (80%) were optimised by most recent follow up (mean 20.0 ± 12.64 weeks, 95% CI 16.16–23.84). A binary logistic regression model was used to assess whether CYP2C19 metaboliser status predicted delayed optimisation, defined as taking more than 12 weeks to reach target dose or not yet being optimised at last follow-up. Due to low numbers, patients with poor (*n* = 2) and ultra-rapid (*n* = 1) phenotypes were excluded. Among the remaining 50 patients, CYP2C19 status (intermediate and rapid vs. normal) was entered as a categorical predictor, using normal metabolisers as the reference.

Intermediate metabolisers had an odds ratio (OR) of 0.63 (95% CI: 0.12–3.19), and rapid metabolisers had an OR of 0.55 (95% CI: 0.11–2.53). The model’s area under the receiver operating characteristic curve (AUC) was 0.57, indicating poor discriminative ability. These findings suggest that CYP2C19 phenotype did not meaningfully predict time to optimisation.

### LVOT gradient

**Fig. 1:**
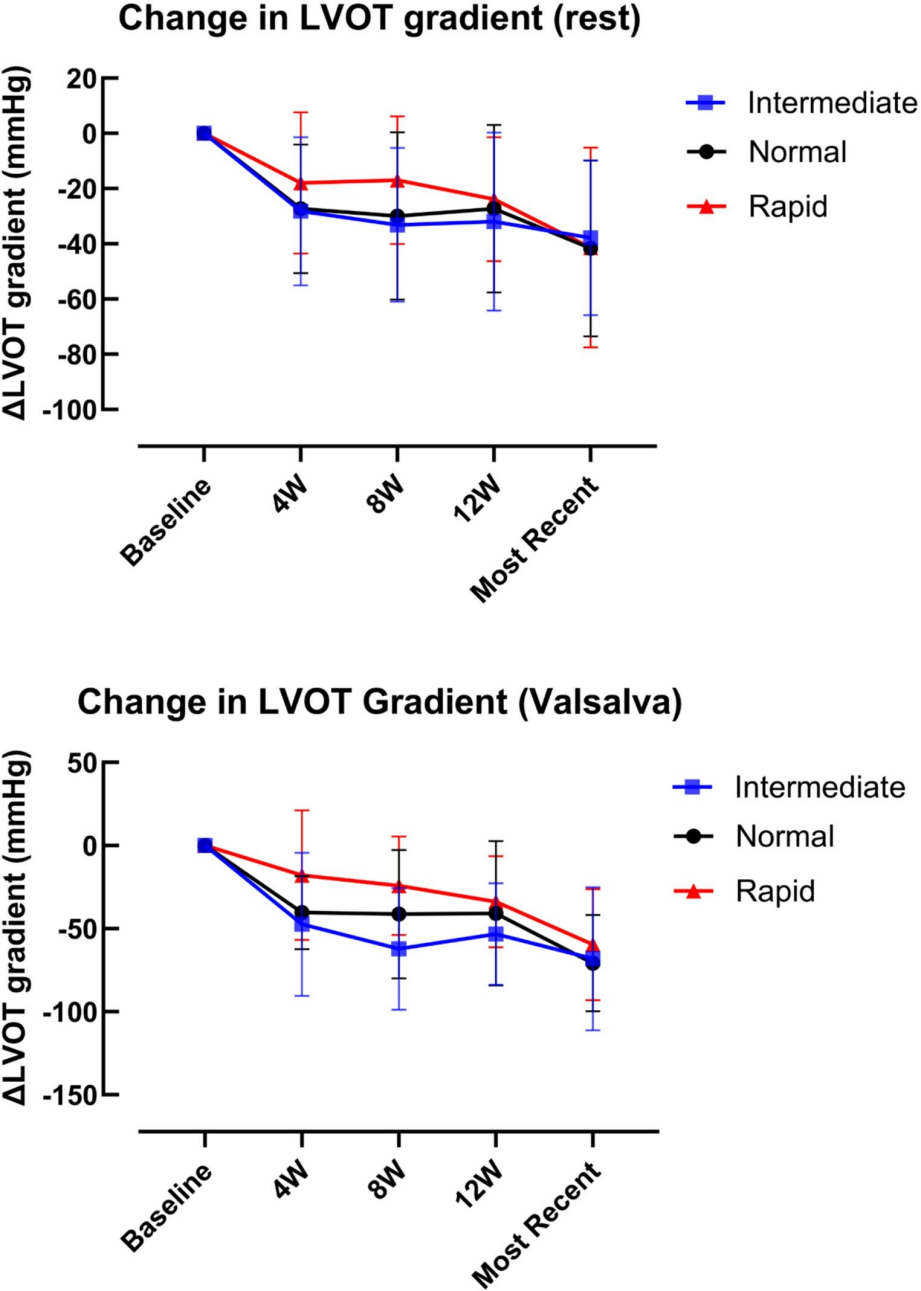
Change in LVOT gradient at rest and with Valsalva manoeuvre in normal, intermediate, and rapid CYP metaboliser groups. Data are mean ± SD. Two-way repeated-measures ANOVA with Geisser–Greenhouse correction demonstrated a significant effect of time for both resting and Valsalva gradients (both *p* < 0.0001), with no effect of CYP metaboliser group (rest *p* = 0.43; Valsalva *p* = 0.38) and no time × group interaction (rest *p* = 0.69; Valsalva *p* = 0.71)

Overall reductions in both peak resting (40 ± 34.37 mmHg) and Valsalva (64 ± 35.23 mmHg) gradients were statistically significant (*p* < 0.0001). Progressive reduction in gradient was observed in rapid, intermediate and normal metabolisers from baseline to the most recent follow-up (Fig. 1). Whilst reduction appeared to be slower in rapid metabolisers, particularly from weeks 4 to 8, this trend was not statistically significant (*p* = 0.43). There was no interaction between group and time (*p* = 0.69), indicating that the degree of gradient reduction was similar across all metaboliser types and not influenced by CYP2C19 status.

Rapid reduction in gradients in the initiation phase (< 20mmHg), leading to dose reduction or temporary cessation of mavacamten, was observed in three patients (5%). Two of these patients were normal metabolisers and one was a rapid metaboliser. These patients are now in the maintenance phase of treatment with optimised gradients.

### Left ventricular ejection fraction (LVEF)

**Fig. 2:**
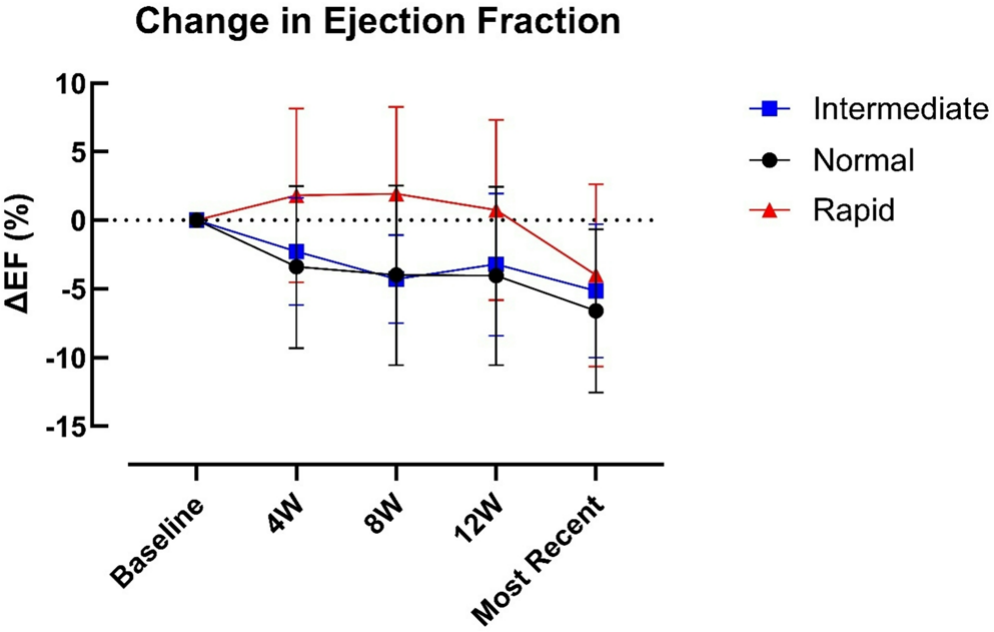
Change in left ventricular ejection fraction (LVEF) during mavacamten therapy across CYP metaboliser groups. Data are shown as mean ± SD. Two-way ANOVA demonstrated significant effects of time (*p* < 0.0001) and CYP metaboliser group (*p* = 0.0091), with no time × group interaction (*p* = 0.21). Tukey post-hoc testing showed higher EF in rapid metabolisers than normal metabolisers at 4 and 8 weeks (*p* = 0.04 and *p* = 0.03, respectively) and higher EF in rapid than intermediate metabolisers at 8 weeks (*p* = 0.006); no other between-group comparisons were significant, and these differences were no longer evident by 12 weeks

Post-hoc comparisons demonstrate that in the initiation phase, rapid metabolisers had a marginally smaller reduction in EF at four and eight weeks compared to normal metabolisers (*p* = 0.04 and 0.03, respectively), and at eight weeks (*p* = 0.006) compared to intermediate metabolisers (Fig. 2). These differences were transient and resolved by 12 weeks.

One patient had a drop in EF < 50% (intermediate metaboliser) during treatment. LV impairment resolved on mavacamten withdrawal, and the patient has since been established back on mavacamten with optimised gradients and preserved LVEF.

### New York heart association class (NYHA)

**Fig. 3:**
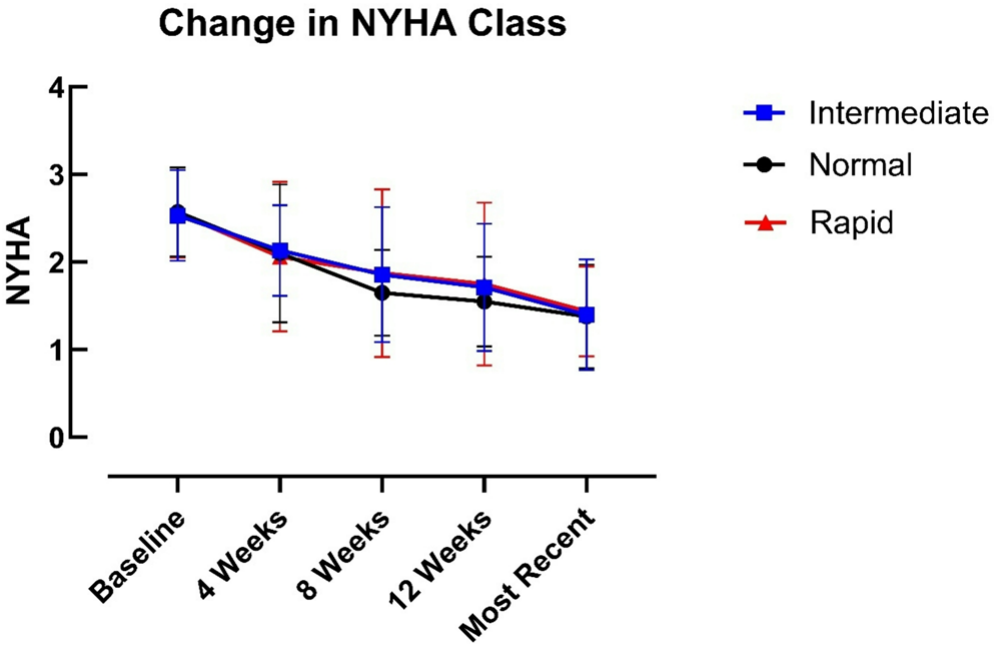
Change in NYHA class from baseline to most recent follow-up in normal, intermediate and rapid metaboliser groups. Data represent median values. Two-way ANOVA showed a significant effect of time (*p* < 0.0001), with no effect of metaboliser group (*p* = 0.63) and no time × group interaction (*p* = 0.99)

All patients had NYHA 2–3 symptoms at baseline. Symptoms improved significantly over time (*p* < 0.0001) in all groups, however there were no significant differences between metaboliser groups at any timepoint (*p* = 0.63) (Fig. 3). Nearly two-thirds of the group had improved to NYHA 1 at most recent follow up (64%).

## Discussion

Our study has shown that *CYP2C19* genotyping does not appear to provide additional benefit in predicting response to mavacamten in intermediate, normal and rapid metabolisers.

Establishing CYP2C19 metaboliser status is currently mandated in Europe and the UK. This differs with North America, where genotyping is not part of the dose initiation protocol, and all patients are commenced on 5 mg once daily. Whilst this is the case, there is still an emphasis on close monitoring and consideration of potential drug-drug interactions [18]. Genetic testing incurs additional time and financial resource for genetic and cardiomyopathy services. Clarifying the utility of this is therefore important. Currently, only determination of CYP2C19 poor metaboliser status results in an alteration to mavacamten dose. It is important to establish if different metaboliser status results in altered responses to the drug or identification of other CYP2C19 variants by extended genotyping adds value.

Variants in the CYP2C19 enzyme affect the terminal half-life (t_1/2_) and hence alter drug exposure of mavacamten [9]. There is established data that supports the lower initiation dose in poor metabolisers, with reduced enzyme function leading to higher drug levels and increased risk of adverse events such as LV systolic dysfunction. Whilst the pharmacokinetic profile of mavacamten suggests that identification of poor metabolisers is beneficial, our study was unable to demonstrate this due to low representation from this group. This may be in part related to our demographic of patients (84% White European, see Table 1). Poor metaboliser status varies by ethnicity, with the lowest prevalence seen in the European population (2.1%) and the highest prevalence in Far East Asians (11.9%) [19]. 

It is worth noting that some of the pharmacokinetic properties of mavacamten may not directly translate to real world practice. Following a single dose of 15 mg mavacamten, AUC increased by 241% and maximum peak concentration increased by 47% in poor metabolisers compared to normal metabolisers [20]. However, as was the case in both EXPLORER and VALOR HCM, the 15 mg dose was proportionally less represented, with only 20% of our cohort established on this in our study [10, 21]. 

Beyond assessing drug efficacy, predicting which patients may be at increased risk of adverse outcomes is important. In our study, adverse events such as development of LV impairment and rapid reduction in LVOT gradient in the initiation phase were rare, occurred in individuals with different *CYP2C19* genotypes, and therefore were not explained by specific variants in the *CYP2C19* gene. While the trajectory of LVOT gradient (Fig. 1) and LVEF (Fig. 2) in rapid metabolisers may support a slower effect of the drug in these patients, which is in line with the pharmacokinetic properties of mavacamten, this did not result in different clinical outcomes by most recent follow up (4–60 weeks). Symptomatic improvement was also consistent irrespective of metaboliser status.

Our study has several limitations. Firstly, our small cohort of majority male, White European patients, and the lack of representation from ultrarapid and poor metaboliser groups limits our ability to extrapolate our results to larger, more diverse oHCM populations. Secondly, though we did not identify rare *CYP2C19* alleles, our cohort was small and our testing approach would not identify non-coding or structural genetic variants. The low adverse event rate also meant that formal statistical comparisons between *CYP2C19* genotypes in this group were not performed. As we could not include the poor metabolisers in our statistical analysis, it is not clear whether *CYP2C29* genotyping and subsequent dose amendment in this cohort translates to a better safety profile of the drug. Whilst the elimination half-life of mavacamten (23 days in poor metabolisers compared to 6–10 days for other phenotypes) supports more cautious dosing in this group, presently our data do not support amendments to the UK and European protocol.

## Conclusion

Outside CYP2C19 poor metabolisers, variations in the *CYP2C19* gene do not appear to effect clinical outcomes in oHCM patients on mavacamten. Whilst a larger and more varied patient cohort is needed to further evaluate this, our study indicates that dose adjustments outside of the CYP2C19 poor metaboliser group are not warranted. Due to small sample size, we were unable to provide definitive evidence as to whether amended dosing in poor metabolisers translates to improved treatment outcomes and a reduction in adverse events. It is therefore unclear whether this practice in the UK and Europe confers any advantage compared to the North American model of care. Nevertheless, the data do not support amendments to the current dosing protocol.

## Supplementary Information

Below is the link to the electronic supplementary material.

Supplementary Material 1

## Data Availability

The data underlying this article will be shared on reasonable request to the corresponding author.
