# Supplementary material for: CYP2C19 genotyping and mavacamten: predicting outcomes in normal, intermediate and rapid metabolisers in obstructive hypertrophic cardiomyopathy
Source: Eur J Clin Pharmacol. 2026 Feb 6;82(3):67. doi: 10.1007/s00228-025-03991-8 (PMC12881021; doi:10.1007/s00228-025-03991-8)
Supplement: Supplementary file 1 — Supplementary Material 1 [file 228_2025_3991_MOESM1_ESM.docx]

**Supplementary table 1:**

Primer sequences used for CYP2C19 genotyping

| **Gene** | **Primer** |
| --- | --- |
| CYP2C191F | TAGTGGGCCTAGGTGATTGG |
| CYP2C19ex1R | CAATGATCTCTTGTAACATTGTACCTC |
| CYP2C19ex2_3F | TTTGAGCCTGTGTGACTGAA |
| CYP2C19ex2_3R | CCCCTGAAATGTTTCCAAGA |
| CYP2C19ex4F | TTTGCTTTTAAGGGAATTCATAGGT |
| CYP2C19ex4R | TTCACCCCATGGCTGTCTAG |
| CYP2C19ex5F | TCTCTTGTCAGAATTTTCTTTCTCAA |
| CYP2C19ex5R | CACAAATACGCAAGCAGTCACA |
| CYP2C19ex6F | CCCTCTCTCACCGCTCCTAT |
| CYP2C19ex6R | AGAAAGGAGAACACTAGCAGCA |
| CYP2C19ex7F | TTCATGTACCCCTGAATTGCT |
| CYP2C19ex7R | TGCACTTCTCTCACCCAGTG |
| CYP2C19ex8F | TGCATGATTACCACTGTTTCTTA |
| CYP2C19ex8R | TGCAGAGAAGGCACATGTAAGT |
| CYP2C19ex9F | TCACCGAACAGTTCTTGCAT |
| CYP2C19ex9R | GATGACGGGTCAGAAGAAGC |
| CYP2C19*17_F | ATCTCTGGGGCTGTTTTCCT |
| CYP2C19 *17_R | ACGTGAAGGCAGGAATTGTT |
